# Supplementary material for: What does it mean to be the main caregiver to a terminally ill family member in Lithuania?: A qualitative study
Source: PLoS One. 2022 May 12;17(5):e0265165. doi: 10.1371/journal.pone.0265165 (PMC9098011; doi:10.1371/journal.pone.0265165)
Supplement: S1 File — (PDF) [file pone.0265165.s001.pdf]

<u>Supplementary File 1. Interview Questions</u>.

Kuznecoviene, J., Butkeviciene, R., Harsisson, W. D., Peicius, E., Urbonas, G., Astromske, K.

Guidelines for questions for relatives caring for people with serious and incurable diseases (English translation of interview questions)

1. Introduction of the project to the interview participant

• Describe the institution in which the research is based and the interviewer.

• Introduce the research aim in relation to qualitative methods of inquiry.

• Ensure participants of their anonymity and possibility to step out of the research. Signing consent to participate in the research.

2. Experiences and daily lives of loved ones caring for patients with terminal illnesses at the end of their lives

• What was your relationship with the person you were caring for?

• What disease was a relative suffering from?

• How did the person you cared for find out the diagnosis?

• Where did they live while being cared for - (in a big city, in a small town, in a village)?

• Where did the person spend the last period of his/her life (home, hospital, residential care)?

• How long did the intensive care period last?

• Please, tell us what was your daily routine as a person caring for a loved one with a serious and incurable disease?

Did someone else take care of the ill person other than you? (For example, social workers, medical professionals, relatives, friends etc.)

3. Ill person's and family care needs

• What do you think was most important to your loved one at the last stage of his/her life?

• What was most important for you, as a caregiver, during the process of caregiving? On what did you focus your attention mostly?

• How were the ill person‘s needs paid attention to and/or met by medical and nursing staff?

• What challenges and difficulties did you experience in meeting the ill person‘s needs?

4. Patient and family participation in making treatment and caregiving decisions

• Did your loved one know about his/her health condition and prognosis? How did he/she get to know about the health situation?

• Were there some situations when important decisions related to treatment/nursing/care had to be made?

• Who participated in making these decisions?

• What was the process? Did professionals invite or include the patient and family members in this process?

• How much and what kind of information about the patient‘s health condition, prognosis, treatment, and nursing care did professionals provide to the patient/family? Was the information provided enough for you in making decisions concerning treatment/nursing/care?

• Did physicians or other staff help you and your loved one in one or another way (sharing their attitudes, giving arguments) to make decisions?

• What did the opinions of professionals ‘and others who were part of this process, mean for the ill person and family? Whose opinion and will were the most important?

• Was it difficult for you and your loved one to make decisions or not?

• What, if any, non-medical professionals discussed and collaborated with the ill person and family or other informal care givers?

• Did you discuss in advance with the ill loved one the situation when he/she would not be able to make decisions?

• How much did family and professionals value and pay attention to the ill person‘s will, needs, expectations?

• Was it possible to fulfil the will of the ill person? Did you meet some challenges? What?

• What issues and how, according to your mind, should professionals discuss, and how should they collaborate on with patient and family? What is your good experience and what are your expectations.

5. Understanding of dignity

- How do you personally understand dignified end-of-life?
- What factors, according to your loved one's nursing experience, ensure dignity at the end of life?

6. Demographical information

- What was the education/profession of your loved one?
- At what age your loved one did die?

IN LITHUANIAN

Klausimų gairės sunkiomis ir nepagydomomis ligomis sergančių asmenų artimiesiems

1. Įvadas

• Trumpas institucijos ir interviuotojo pristatymas.

• Projekto tikslas, trumpas interviu, kaip kokybinių tyrimų metodo paaiškinimas.

• Anonimiškumo užtikrinimas, galimybė atsisakyti dalyvauti tyrime, sutikimo dalyvauti tyrime pristatymas.

2. Slaugančiojo artimojo patirtis ir kasdienybė

• Koks buvo Jūsų ryšys su slaugytu žmogumi?

• Kokia liga sirgo artimasis?

- • Kaip Jūsų slaugytas artimasis sužinojo diagnozę?
- • Kur gyveno artimasis kai buvo slaugomas - (didmiestyje, mažame mieste, kaime)?
- • Kur buvo slaugomas artimasis paskutinį gyvenimo etapą namuose, ligoninėje, globos įstaigoje?)
- • Kiek truko intensyvios priežiūros laikotarpis?
- • Papasakokite, kokia kasdienybė slaugant sunkia ir nepagydoma liga sergantį artimąjį?
- • Kas dar padėdavo šeimai rūpintis sergančiuoju? (socialiniai darbuotojai, slaugytojai, kiti giminės ir t.t.?)

3. Sergančiojo /šeimos poreikiai

• Kas, jūsų nuomone, sergančiajam buvo svarbiausia paskutiniame jo/jos gyvenimo etape?

• Kas Jums buvo svarbiausia santykiuose su slaugomu ir slaugymo procese? Kam skirdavote daugiausia dėmesio, kas Jums atrodė svarbiausia?

• Ar į paciento poreikius atsižvelgdavo, ar stengęsi juos atpažinti slaugos specialistai, medicinos personalas?

• Ar buvo sunkumų norint patenkinti jo/jos poreikius? Kokie su tuo susiję sunkumai, problemos?

4. Paciento ir šeimos dalyvavimas gydymo/slaugos/priežiūros procese

• Ar sergantis asmuo žinojo savo sveikatos būklę ir prognozes? Kokiu būdu jis/ji sužinojo?

• Ar buvo situacijų, kai reikėjo priimti svarbius su gydymu/slauga/priežiūra susijusius sprendimus?

• Kas dalyvaudavo priimant sprendimus?

• Kaip vykdavo sprendimų priėmimo procesas? (ar specialistai įtraukdavo į sprendimų priėmimą pacientą ir jo/jos artimuosius?)

• Kiek ir kokios informacijos apie ligonio būklę, perspektyvas, galimas gydymo ir /ar slaugymo strategijas) suteikdavo medikai? Ar informacija buvo, Jūsų požiūriu, išsami, pakankama, kad priimtumėte su gydymu ar tolesne slauga susijusius sprendimus?

• Ar gydytojai kaip nors padėdavo priimti sprendimus (išsakydavo savo požiūrį ir argumentus) ar tai reikėjo padaryti patiems?

• Ar artimiesiems ir pacientui buvo svarbi specialistų ir visų kitų dalyvaujančių procese asmenų nuomonė, kiek į ją buvo atsižvelgiama? Kieno nuomonė ar valia buvo svarbiausia ? (socialinis pripažinimas)

• Ar sunku būdavo priimti sprendimus?

• Kokiais dar klausimais, be gydymo, su pacientu ir šeima tarėsi ir bendradarbiavo specialistai?

• Ar buvo iš anksto su artimuoju kalbama, kaip elgtis, kai pats negalės priimti sprendimų?

• Kiek artimieji ir gydytojai bei medicinos personalas atsižvelgė į slaugomojo valią, poreikius, lūkesčius?

• Ar ir kiek buvo įmanoma išpildyti artimojo valią? Su kokiomis kliūtimis susidūrėte?

• Kokiais klausimais ir kaip, artimųjų nuomone, turėtų su pacientu ir šeima bendradarbiauti (tartis) specialistai: geroji patirtis ir lūkesčiai?

5. Orumo samprata

• Ką reiškia Jums ori gyvenimo pabaiga?

• Kas, Jūsų požiūriu ir remiantis Jūsų artimojo slaugymo patirtimi, užtikrina orumą gyvenimo pabaigoje ir kokios kliūtys tam trukdo?

6. Demografiniai klausimai

• Koks buvo slaugyto artimojo išsilavinimas?

• Koks buvo slaugyto artimojo amžius?
