# Supplementary material for: What does it mean to be the main caregiver to a terminally ill family member in Lithuania?: A qualitative study
Source: PLoS One. 2022 May 12;17(5):e0265165. doi: 10.1371/journal.pone.0265165 (PMC9098011; doi:10.1371/journal.pone.0265165)
Supplement: S2 File — https://doi.org/10.5281/zenodo.6334994. (DOCX) [file pone.0265165.s002.docx]

**Supplementary File 2**

**Translated samples of original Lithuanian interview texts**

1. **Participant, a woman, 37, cared for her mother**

INTERVIEWER: Before taking your mother home she spent some time at hospital…

PARTICIPANT: …I don’t think anyone will care as much as I do. Even the doctor talked about it. And to be done with love, not like there in hospital. Mom paid 5 euros each day for changing her sheets… And she payed extra money for nurse assistance to wash their heads. I don’t blame because I know what the loads are, but then I think who will take care if not me. So, fresh air, washing ............. When I took her home from hospital, I put her to my bed, but I saw the big pain she suffered. So, I found announcement in internet about functional bed, special mattress and rented all things needed.

..........

INTERVIEWER: Did anyone help you, come to see you?

PARTICIPANT: No one. I called the Maltese. They say maybe in half a year there will be an opportunity. I paid the bribe to the doctor of the family to come at least. Such a Soviet kind of thinking. For someone to come.

INTERVIEWER: No one suggested?

PARTICIPANT: Didn’t offer anything, every time with bribes. Good thing we had money to buy all ointments, pampers ...

INTERVIEWER: And the doctor didn't prescribe the pampers?

PARTICIPANT: No. I found out about compensation of diapers quite by accident when I went to the pharmacy to get fentanyl and the pharmacist told me. Then I got angry...

INTERVIEWER: You mentioned your neighbor…

PARTICIPANT: Thank God, we had a neighbor who was a nurse, retired. I asked her to come to do intravenous injections when Mom was sick with pneumonia.

..........

PARTICIPANT: My Mom was afraid to stay alone at home. So, it became more and more difficult psychologically. Especially in nighttime. She asked me to come and be with her. I couldn‘t sleep normally ... I felt I was getting crazy... 24 hours at home with her.

1. **Participant, a granddaughter, 49, cared for her grandmother**

INTERVIEWER: You mentioned that you would not take her [grandma] even to private nursing homes ... How did you decide that only the family would take care of her?

PARTICIPANT: I lived with my grandmother until adolescence ... She was very scared to die in the hospital, kept asking for home. My mother, her daughter, was also sick, so we advised that I take [grandmother] to me and, in exchange for my mother, take care of her. I can‘t imagine strangers doing all these intimate things to my grandma ... I think that all people expect to die at home.

INTERVIEWER: You saw her needs from your own point of view?

PARTICIPANT: I think I personally would like that ... I would like it that way, I think the other person too. I would like to be at home, my children, my grandchildren ... That no strangers would change pampers for me ... I can’t believe a person would want to go to a nursing home if he has loved ones.

1. **Participant, a granddaughter, 48, cared for her grandmother**

INTERVIEWER: We yourself work at hospital...What about dignified end-of-life at hospital?

PARTICIPANT: ...Imagine one day, I was all day at hospital with my grandmother and around those people whose relatives don't come and don't even bring water to drink ... Imagine brings food ... Maybe they can't ... . One man came to visit his mom, so there was a conflict that the mother was not cared for. Says I can’t, I work and I can’t take care myself. Finally, when I do it myself, I do it with love, I ask if it’s convenient for you or if it doesn’t hurt you. And there the nurse came in with a trolley in two, the blanket was torn off, so technically it quickly changed the pampers, there it was liked or disliked. Two minutes of work. I think about how that person feels.

..........

INTERVIEWER: It seems that your were the only one taking care of grandma…

PARTICIPANT: My mom spent time with grandma, being together and speaking with her, but all nursing was on me. I am not afraid of that, I know what and how to do, even if Grandma was resisting. Mom said, you are bothering her too much, but I explained that I am doing what I have to do. We will have problems later if we don’t do all cleaning and washing.

1. **Participant, a daughter, 47, cared for her father**

INTERVIEWER: How did hopital staff reacted to his [father‘s] behaviour?

PARTICIPANT: They [staff] should know [how to behave when a patient has delirium and screams], this is not the only case. Well, it was everywhere. Some nurses do medical injections, tries to calm down but others shout at him. Staff has to know about this condition, they are trained.

..........

INTERVIEWER: To invite the priest…Did you discussed that with somebody from your family?

PARTICIPANT*:* My family handed over everything related to his care to my hands. But that surrender also means responsibility. They’re about "you’re in medicine, you know everything best, you’re in order."

INTERVIEWER: How did you feel about that?

PARTICIPANT: ... I’m used to it. It has long been my responsibility. I accepted it as normal ... It goes without saying that it was me.

1. **Participant, a daughter, 59, cared for her father**

INTERVIEWER: Have you thought about the hospital, the nursing home, when the situation started to get worse?

PARTICIPANT: My children won‘t take care of me in the way I did ... The idea of putting your parents into nursing hospital never would come to the mind of our generation. Unless your mutual relationship is broken. But for my children it wouldn‘t be any problem.

1. **Participant, a daughter, 52, cared for her mother**

INTERVIEWER: You had contacts with oncologists …

PARTICIPANT: I used to call oncologists and ask should I offer food to her when she refuses. Sometimes I got very angry because Mom refused to take medicine, I didn’t know what to do. I asked her physician how should I speak to Mom to convince her to take it. And what to do in case of pain? What medicine would help? You know, everything is new, you don’t know what to do.

..........

INTERVIEWER: As I understood you took your Mom to chemotherapy day care day?

PARTICIPANT: She was angry by the fact that there were no places in the ƒ day care clinics. The beds there did not meet patients’ needs. She said we’re not treated like people.

..........

INTERVIEWER: What was the time, if any, when you decided she no longer needed those drugs?

PARTICIPANT: She herself decided, you know, but she didn't tell me. She didn’t take medication for a couple of weeks. We realized when I found the pills to start... She seemed to feel tired and unable to ...

INTERVIEWER: But she didn't talk to you about it?

PARTICIPANT: No.

INTERVIEWER: I thought maybe she felt it was important for you that she takes those drugs.

PARTICIPANT: I think she saw how we went through it so that she would only take those drugs to improve. And herself, apparently, she felt. She kept saying to me, "Nothing, everything will be fine."

..........

INTERVIEWER: You took care, only by yourself?

PARTICIPANT: I was still working at that time and my brother helped me at home to take care of our mother. Mother kept saying “Don‘t leave your job.” I took three weeks of vacation, and at the end of vacation I said that I will try to prolong vacation by asking for unpaid days. She repeated several times: “Please, don’t give up your job.”

1. **Participant, a wife, 57, cared for her husband**

INTERVIEWER: Your husband died at home?

PARTICIPANT: At hospital. When his temperature got to 40 then we called an ambulance. We once thought he had a heart attack because he wasn't breathing... The ambulance came and said “this is oncology, why didn’t you tell us about that?” But when you call they don’t ask about that, only some certain questions need to be answered. Took us to the clinic, lying there at the reception, but there for about 5-6 hours. Dripping from pain, but that lying down. After 3-4 hours new sores appear. And the place still doesn’t always allow me to sit there… My husband talked to the surgeon who operated on him... I haven't talked to him. [surgeon offered new examinations] Three-four days before examination they do not give food and he gets weak. Although they [the doctors] say there's nothing left to do. And this examination is like mini operation. Three or four days he can not eat, and you can‘t understand why. So I took him home and did a drip myself.

..........

INTERVIEWER: What was the most important things for your husband at the end of his life?

PARTICIPANT: He always took a phone and computer to the hospital. He corresponded with the whole world... but not in intensive care unit.......... Maybe staff was afraid he would record their conversations? They found a phone under his pillow and made a terrible noise shouting at him.

..........

INTERVIEWER: You were near your husband around the clock ...

PARTICIPANT: My husband did not have that feeling of guilt/inconvenience that he needed to be taken care of. He would say you are a saint, but as if it was necessary.

..........

INTERVIEWER: What about you experience paying unofficial money for staff?

PARTICIPANT: We gave [money] everywhere. For everyone. You think differently when you encounter it [system]. Definitely have to give.

1. **Participant, a wife, 46, cared for her husband**

INTERVIEWER: You were together with your husband at the moment of diagnosis?

PARTICIPANT: Hematologist first invited me and gave his diagnosis. And asked if she could speak about that with my husband.

..........

INTERVIEWER: When your doctor told you about the transplantation, did you ask and discuss about other options?

PARTICIPANT: Oncologist asked me what treatment we chose: traditional or alternative. I knew what traditional means; they explained, but I wasn’t sure about alternative. So I was looking for contact, finding people who maybe knew more.

..........

INTERVIEWER: What about your experience taking care at home?

PARTICIPANT: I learnt myself to do intravenous infusion at home. Every day and night I did those infusions… The only thing I didn’t know how to do was how to insert a urinary catheter.

1. **Participant, wife, 68, cared for her husband**

INTERVIEWER: Did your husband know the true of his health?

PARTICIPANT: He did not understand, he didn‘t know.

INTERVIEWER: But the doctors explained to you?

PARTICIPANT: Explained. Then I went home and consulted with my daughters [decided not to tell]...

INTERVIEWER: He did not ask himself?..

PARTICIPANT: He himself did not ask anything... We didn’t know if he [husband] understood his health condition. I didn’t want to ask if he knew what is going on. What if he asks “what is going on with me? What to say? Keep lying?

1. **Participant, son, 48, cared for his father**

INTERVIEWER: I return to the question about the care at hospital… How was it in your case? What was good and what was missing?

PARTICIPANT: They took care of him at the hospital. Diapers always replaced when needed. We paid additional money for nurses and assistants. And they really took care of him. He loved to shave and be tidy. Even on the last day. I brought him what he wanted to eat. We talked, we talked to keep that humanity.

..........

INTERVIEWER: Didn't you leave him alone at hospital?

PARTICIPANT: We left overnight. He was well cared for.

..........

INTERVIEWER: As I understood you tried to spend as much time as possible with your father at hospital…

PARTICIPANT: Well, I saw how they [nurses] treated patients [at hospital] ... those who don’t have somebody visiting him in the ward. How to put it, ... very formally. For example, they bring food, put it on a cabinet next to bed and leave. In some time they come again, say, “You didn’t eat anything,” and take it out. But the patient in that situation can’t eat by himself, he needs somebody’s help.

1. **Participant, daughter, 50, cared for her mother**

INTERVIEWER: So, you were referred to palliative care….

PARTICIPANT: When we arrived at the hospital, she really wanted peace. But women in the ward seemed to be in pain... One woman was screaming very much.......... “Oh, my God, how much can she scream?” Also that she had to wait for examination. How long can you wait here…?

..........

PARTICIPANT [telling about the experience of the last days in palliative care unit]: I understood it wasn’t the first time she [nurse assistant] used those rude words screaming at her. Mom started to apologize to me for getting wet in bed.......... But these rude words were a shock for Mom. I got lost, started trembling ... I didn’t know what to do. I called to my brother, I wanted to ask him what we should do. But we have never left her alone at hospital since that day.

1. **Participant, daughter, 41, cared for her father**

INTERVIEWER: So, you father died in the palliative care unit?

PARTICIPANT: We were referred to palliative care right after hospitalization in the oncology unit [different hospitals]. But when we arrived they said there were no places. We tried to explain that oncology unit sent us and had called them before. They said it means someone was already hospitalized in that place. I said “No, we have to have a place.” It is God’s grace that just at that moment one woman took her mother to a private nursing home and we were left with that place, so we got a single ward [very difficult to get it].

..........

INTERVIEWER: What about the family physician’s help in all this process?

PARTICIPANT: We had to come to the outpatient clinic to meet the family physician ourselves [the patient was no longer able to walk] because of the test needed… While visiting at polyclinic, the family physician saw how bad the situation was, but she didn‘t react. She knew [father’s] real situation. I kept asking to hospitalize him since March but nothing happened...

**13. Participant, a woman, 55, cared for a husband**

INTERVIEWER: What was the most difficult for you when you cared for your husband?

PARTICIPANT. End-of-life care is clearly the most difficult period, because you are completely alone, because there was no help from official medicine, neither psychological nor social, absolutely none. It wasn’t like they would say, for example, you’ll have a hard time. If you need medication ... painkillers, contact this or this specialist. If you are working, then maybe you need someone to be with the patient, then contact there, they will help you find care. Whether or... let’s say, maybe you’d like a psychologist or a clergyman to talk to the patient.......... There were no such questions and suggestions at all.

..........

INTERVIEWER: And what do you think if there had been such comprehensive help from the beginning? Do you see that the last period of his life would have been any different?

PARTICIPANT: [...] I think so. I think the news is different because I would then, as you say, have a lot of activity to do without purpose. I am not talking about the need to organize there for special needs, to run around the institutions, to carry paper, to wait in lines. I didn’t take advantage of those needs until the papers found their way to me; the man was already dead. And the needs were set at 56 euros. What for 56 euros, what needs can you meet for a person? Even diapers cannot be bought, nothing, that is it. And then apparently when it would have been before, that cozy, because there is a set period of time over which a person can get worse, he can’t get worse in a day or two, apparently only a month or two is allowed!. In a word, as you say, there is no focus on the person or the nurse. There are some acts of law somewhere, but they are somewhere in the sky. To the particular person who needs them, they do not appear; they have to look out for themselves, and in order to look out, they still have to know where to look, that is. This is what I think, as soon as such a diagnosis occurs in a person, he must get comprehensive information on where and what questions he should turn to. It is then possible to plan that time in the best way possible for both the patient and the nurse, because if the nurse is ill, the patient is ill.

..........

INTERVIEWER: You said that finally you were left alone to care for your husband. Why, do you think?

And the close people who used to interact with him have now backed down. And only then did I realize they didn’t know what to do in that situation. They don’t know, they’re afraid of pain, they’re afraid to touch pain, they don’t know what to talk about, they just put it in the bushes, that’s to say nowhere. For me, that suffering is my own and I cannot escape from it and I do not want to escape. It is good for me to do good to a loved one, to do as he needs to, as I understand, how we feel we need to do. This is my situation. And for others there is no need to worry, a burden they do not know how to lift, do not know what to do with it.

**14. Participant, a woman, 51, cared for a husband**

INTERVIEWER: What is you experience of caring for your husband?

PARTICIPANT: I had to spent a lot of time doing such… I had no time to be with him… for example, the bed, without that functional bed it would be impossible because I personally cannot do physical work myself, I can neither bend over nor lift hard. That’s without a functional bed is then all has to be done. You need to either hire someone or admit them to some kind of hospital.

..........

PARTICIPANT: I hired a functional bed, I rented it myself, no one told me that you can [get help], or even that “I will write to you, you can rent, you can borrow,” that there are services that lend all those things. No information on this. That's what I find myself on the internet and then I run and look for. I also rented an oxygen machine myself and that was genetically expensive one. Then only as my mother needed to be cared for did I discover that it was almost half as cheap to rent. But I also rented it myself, I didn't know, I didn't have any such technical support information. That's how much extra money was spent where you might not have it.

..........

But I accidentally talked to a X about palliative care and she says, look, I have established such an enterprise for palliative care and... then, we can arrange the paperwork and it is free, it is state reimbursed and we can try to help you. And so, these people who came through personal contacts, were of gold value.

**15. Participant, a woman, 55 cared for mother**

INTERVIEWER: What does it mean to you to take care of a seriously ill loved one? What are your experiences?

PARTICIPANT: But really, where to put a person who, well, you really cannot have him, because we work too and you cannot take him, you do not solve that problem, because you come back in the evening and who will take care of her all day? And that anxiety was that a long time ago, that she was walking and going to the store and everything, but we've already seen everything that is coming, what everything will be someday, so that you need it and you don't know where to get it, how to solve anything.

..........

INTERVIEWER: Why do you think you have been left alone to care her?

PARTICIPANT: people are not taught to be with another person’s suffering. It’s so.

**16**. **Participant, a woman, 65, who cared for her sister**

INTERVIEWER: What was most difficult for you in nursing your sister?

PARTICIPANT: When she was released home from the hospital… Those first days. I only remember a very scary thing when you come and think, Lord, send me someone to show me how to do something here. Where to find any information? Well, that then I called her doctors. I was visiting her after going to the family doctor. I tell the doctor, provide a woman to show me [how to do things] and that I shake hands so I know what to do. And in general, how do I take care of her? Who can help me? Well, no, no information ..

..........

INTERVIEWER. So, did she spend her last days at home?

PARTICIPANT: And nursing hospital, if without any such attempts to agree in some way with administration, then it puts you in line and you wait in line until someone from there dies. It’s full absolutely… [until then].

**17**. **Participant, a woman, 61, who cared for her husband**

INTERVIEWER. What was the most important at that time for you?

PARTICIPANT: To meet his needs of course… To organize all of it. But you know… Actually those so called physical needs firstly. Because I did not know what to do…

.........

INTERVIEWER. And what about yourself?

PARTICIPANT: It was difficult for me and maybe I feel some kind of reproach that I said, oh how tired I am, because I was just very tired, and I didn’t hold back.......... Probably he didn’t feel dignified at moments like that.......... I don’t know, maybe.

..........

INTERVIEWER: Who else cared for him?

PARTICIPANT: At the beginning his son…But usually after a while it remains only one person who is caring, the main one. Because the whole family will not take care of loved one, because it is too difficult for them, and the habits need to be known. So one person remains. And that loved one also wants to be with that one person. He doesn’t really want to see other people. My husband didn’t even let other people take care of him. That’s right, I still draw the following conclusion from my experience in the end - there is only one person who cares. The main person remains, everyone else comes, visits, leaves.

**18. Participant, a women, 57, who cared for husband**

INTERVIEWER: And how does communication with the doctors run?

PARTICIPANT: There are exceptions, of course, but the tendency is that there is no respect for the person who came to the doctor. So, that's what I'm saying, you can't call that doctor anytime…. No, the doctors do not like you're calling when you really need it. It would be good if the family doctor was someone you can trust, you can call, call him at any time, consult…In a word, there is no respect for a person - neither for that patient nor for the caregiver.

**19. Participant, a man, 65, who took care of his wife**

INTERVIEWER: Why did she not want to accept a visitor?

PARTICIPANT: Because people come and knock the situation even more out of balance. And even those glimpses of pity, words of pity, movements of pity like, say, show that everyone already knows that the person is already doomed. And then the person closes himself. Finally, she didn’t want to communicate with anyone. Her will was for no one to visit.
