# Supplementary material for: What does it mean to be the main caregiver to a terminally ill family member in Lithuania?: A qualitative study
Source: PLoS One. 2022 May 12;17(5):e0265165. doi: 10.1371/journal.pone.0265165 (PMC9098011; doi:10.1371/journal.pone.0265165)
Supplement: S3 File — (DOCX) [file pone.0265165.s003.docx]

| **Supplementary File 3.**  **Progression from sample of interview text to codes, categories, and theme** | | | |
| --- | --- | --- | --- |
| **Sample Quotations from interviews leading to codes 🡪** | **Codes leading to categories 🡪** | **Categories leading to theme 🡪** | **Theme 1** |
|  |  |  |  |
| In a word, there is no respect for a person - neither for that patient nor for the caregiver. There are some law somewhere, but they are somewhere in the sky, to the particular person who needs them, they do not appear, they have to look for themselves, and in order to look, they still have to know where to look, that is. (Rima) | 1. No professionals asked questions about needs  2. Laws *are “*in the sky*”*  3. No suggestions whom to contact | ***Category 1.***  **Lack of awareness of public care services** | **Inaccessibility and mistrust of public care services**  ***(Theme built from categories. It covers entire column.)*** |
| End-of-life care is clearly the most difficult period, because you are completely alone, because there was no help from official medicine, neither psychological nor social, absolutely none.... It wasn’t like they would say, for example, you’ll have a hard time… if you need medication ..painkillers, contact this or this specialist. If you are working, acha you are working, then maybe you need someone to be with the patient, then contact there, they will help you find care. Whether or … let’s say,…, maybe you’d like a psychologist or a clergyman to talk to the patient. ..There were no such questions and suggestions at all (Orinta) | 4. You have to know and search yourself |  |  |
| That’s what I think, as soon as such a diagnosis is said for somebody, he needs to get comprehensive information on where and what questions he should turn to. It is then possible to plan that time in the best way possible for both the patient and the nurse ’(Rima ) | 5. Immediate and comprehensive information after diagnosis |  |  |
| I rent a functional bed, I hired it myself, no one told me that you can, I'll tell you, you can rent, borrow, that there are services that lend those things to everyone. No information on this. That's what I need to find on the Internet and then I run and look, so I rent the oxygen machine myself and that functional bed and genetically expensive. Then just as my mother needed to be cared for, I discovered that it was barely half as cheap as renting. But I also didn't know, I didn't have any such technical support information. It's still such extra money spent where you might not have them.' | 6. Information necessary for care planing/ information on nursing facilities |  |  |
| I accidentally talked to a former student about palliative care and she says, look, I have established such an enterprise for palliative care and she says, look, I have established such an enterprise for palliative care and then, we can arrange the paperwork and it is free, it is state reimbursed and we can try to help you. And so, these people who came through personal contacts, were of gold value.  We learned from somewhere...  We accidentally heard people talking about these things [information needed] | 7. *G*old value of personal contacts  8. Information learned by accident |  |  |
| I found out about compensation of diapers quite by accident when I went to the pharmacy to get fentanyl and the pharmacist told me. Then I got angry...  We didn’t know for what support we could apply. Only later we realized. | 9. No knowledge about compensations or benefits |  |  |
| I was looking for advertisements [functional bed] in internet and newspapers. | 10. Looking at public newspapers for material needs |  |  |
| When we came back home ... I only remember a very terrible thing when you come bach with your ill loved one after hospital... and thought of the Lord .. somebody to explain or show someone how to do things here. Nu this, then I call her doctors I went to her family doctor. I say doctor, give me a woman to show me and that I'm shaking hands so I know what to do. | 11. Lack of information on how to care |  |  |
|  | | |  |
| And nursing hospital, if without any such attempts to agree in some way with administration, then it puts you in line and you wait in line until someone from there dies. It’s full absolutely [until then].  We were referred to palliative care right after hospitalization in oncology units [different hospitals]. But when we arrived they said there were no places. We tried to expalin that oncology unit sent us and called them before. They said it means someone was hospitalized in that place. | 12. No places in palliative care facilities | ***Category 2.***  **Unavailability of public health care services** |  |
| There are exceptions, of course, but the tendency is that there is no respect for the person who came to the doctor. So, that's what I'm saying, you can't call that doctor anytime,... No, the doctor's not like you're calling when you really need it. It would be good if the family doctor was someone you can trust him, you can call him, call him at any time, consult (Rita*)* | 13. Family doctor is not easily available |  |  |
| But there were no places in that unit, but he asked the person he knew some to help with hospitalization in intensive care unit | 14. Obtaining service through informal contacts |  |  |
| ...he was hopitalized after my call to some people who new physiscians in that hospital. I paid staff, for the ward, for the services.  Mom paid 5 euros every day for changing the sheets. And she paid extra money to the nurse asistant for washing her head.  If it hurts there, if it's reddened, that someone from the professional nurses would come .... but you know, they say there's no enough nurses.... When nurse comes... I gave 10 euros, next time 10 euros and finally it is big money...  We gave [money] everywhere. For everyone. You think differently when you encounter it [system]. Definitely have to give*.*  I paid the bribe to the doctor to come and visist Mom at home. Such a Soviet kind of thinking... I was scared, paying for not leaving us at all. | 15. Bribes or informal (unofficial) payment for services: (shortening the waiting time, expecting more attention and better services) |  |  |
|  | | |  |
| We took her to the hospital admission department and I ask if they have a functional bed. They say that they don‘t have any functional beds. The deparmnet full of patients, we thought it would be bad at all for her there.. I had to make a decision on what to do. And I say we will take her back home.  In our nursing hospitals, I don’t think it’s an dignified death. When my grandmother’s sister was hospitalized I remember the smell of urine right after you enter the hospital.  When we arrived at the hospital, she really wanted peace. But women in the ward seemed to be in pain...One woman was screaming very much.... Oh, my God, how much she can scream. Also that she had to wait for examination.How long can you wait here. | 16. Negative hospital environment (bad smells, people in pain in the same ward, no arrangements needed) | ***Category 3.***  **Patients, family and friends view services negatively** |  |
| We waited for about four hours (at the pain clinic). The wait was very complicated. Those pampers, everything goes, rot.... Again [after filling in the questionnaire] we waited for a long time about 4-6 hours.  ...you won't sort everything out in a month [time to prepare the doccuments for benefits]. I remember how much time my colleague spent for all the paperwork. How long they had to wait for that help to be given.  We had to come to outpatient clinic to meet family physician ourselves [the patient was no longer able to walk] because of the test needed. | 17. Bureaucratic approach |  |  |
| And in the institutions, it’s just that the hypnotics are injected so that the patient doesn’t ask for anything  Three-four days before examination they do not give food and gets weak. Although they [the doctors] tell there's nothing left to do. And this examination is like mini operation. 3-4 days can not eat and you can‘t understand why. So I took him home and did a drip myself.  While visitng at polyclinic, family physician saw how bad the situation was but she didn‘t react. She knew his real situation. I kept asking to hospitalize him since the March but nothing happened... | 18. Distrust of treatment and professionals‘ attitudes |  |  |
| He always took a phone and computer to the hospital. He corresponded with the whole world... but in internsive care unit... Maybe staff was afraid he record their conversations? They found a phone under the pillow, and made a terrible noise shouting at him. | 19. Hospital rules: no phones |  |  |
| But after that [the nurse's assistant shouted at the patient], we haven't left her alone.  They [staff] should know [how to behave when a patient has delirium and screams], this is not the only case. Well, it was everywhere. Some nurses do medical injections, tries to calm down but others shout at him. Staff has to know about this condition, they are trained.    She [patient] was angry by the fact that there were no places in the chemotheraphy day care clinics. The beds there did not meet patients‘ needs. She said we’re not treated like people. | 20. Disrespect for patient |  |  |
