# Supplementary material for: What does it mean to be the main caregiver to a terminally ill family member in Lithuania?: A qualitative study
Source: PLoS One. 2022 May 12;17(5):e0265165. doi: 10.1371/journal.pone.0265165 (PMC9098011; doi:10.1371/journal.pone.0265165)
Supplement: S4 File — (PDF) [file pone.0265165.s004.pdf]

S2 File. Themes, Categories, Quotes (Temos, kategorijos, citatos)

Themes	Categories	Quotes (in Lithuanian)
**1.Paliaityvios pagalbos paslaugų neprieinamumas ir nepasitikėjimas jų kokybe**	1a. informacijos apie slaugos paslaugas stoka	Tai (slaugymas artimojo gyvavimo pabaigoje) yra aišku pats sudėtingiausias laikotarpis, todėl, kad na esi visiškai vienas, nes iš oficialiosios medicinos nebuvo jokios pagalbos nei psichologinės nei socialinės, jokios absoliučiai. (...) Tai nebuvo taip, kad Jums bus sunku, jeigu jums reikalinga dėl vaistų nuskausminamųjų, kreipkitės vat į šitą ar šitą specialistą. Jeigu Jus dirbate, acha jus dirbate, tai gal jums reikėtų, kad pabūtų su Jumis, tai jus kreipkitės ten, Jums padės surasti priežiūra. Tai ar ar ar sakykime, ten gal jus norėtumėt, kad su ligonių pasikalbėtų ar psichologas ar, ar, ar dvasininkas irgi tokių klasuimų ir pasiūlymų nebuvo visiškai. (Žmona, 55 m.)<br><br>Vienu žodžiu, tokie kaip sakyt, dėmesio žmogui nėra jokio nei tam pacientui nei slaugytojui. Yra kažkur kažkokie aktai teisės, bet jie yra kažkur danguj, prie to konkretaus žmogaus kuriam jų reikia, jie neatsiranda, jie pats turi ieškoti, o kad ieškoti, dar turi žinot kur ieškoti, tai va. Tai aš manau, jeigu tik atsiranda tokia diagnozė žmogui nustatoma, jis turi gauti visapusišką informaciją kur ir kokių klausimų jis turėtų kreiptis. Tada galima ta laiką planuoti taip, kaip galima geriausiai ir pacientui ir pačiam slaugytojui. (Žmona, 57 m.)<br><br>Netyčia išsikalbėjau su viena tokia buvusia studente apie paliatyvią slaugą ir sako, va aš turiu įsteigusi tokią įstaigą paliatyviosios slaugos ir tada mes galim, sutvarkyti dokumentus ir tai yra nemokama valstybės kompensuojama ir mes galim pabandyti padėti. Ir va, šitie žmonės, kurie atėjo padėtį be tik asmeninių ryšių pagalba netyčia išsikalbėjom taip yra ir tie žmonės na buvo aukso vertės. (Žmona, 51 m.)<br><br>Aš nieko nežinojau apie kompensaciją už sauskelnes ar kitas išmokas ... Apie tai [sauskelnes] sužinojau visai atsitiktinai, kai nuėjau į vaistinę pasiimti fentanilio ir vaistininkas man pasakė. Tada aš supykau... (Dukra, 37 m.)

		Apie ligą, apie ligą. Ne, jau čia nėra tokio spagalbos... tikrai niekas nepasiūlė ką daryti, kaip daryti, Čia jau visiškai savarankiškai tu galvoji ką daryti. (Sesuo, 73 m.)<br><br>Funkcinę lovą samdžiau, pati nuomavau, niekas man nepasakė, kad tu gali, tau aš išrašysiu, tu gali nuomuotis, skolintis, kad yra tarnybos, kurios skolina tuos dalykus visus. Informacijos jokios apie tai. Tai aš pati reikia susirandu internete ir tada bėgu ir nu ieškau, tai ir deguonies aparatą pati nuomavausi ir ir tą funkcinę lovą ir genetinai brangiai. Paskui tik tai, kaip mamai reikėjo slaugyti atradau, kad vos ne per pus pigiau galima buvo nuomotis. Bet irgi nuomavau pati irgi nežinojai, jokios tokios vat socialinės kažkokios techninės pagalbos informacijos jokios neturėjau. Tai dar vat tokių papildomų lėšų išsileido, kur galbūt galėjai ir neturėti jų. (Dukra, 45 m.)<br><br>Tai va, aš ir manyčiau, kad tas šeimos gydytojas, tai toksai ir būtų taip kaip skamba šeimos gydytojas, šeimos, vos ne narys. Aišku, jis kartu negyvena, kad nebūtų problema vat pasiskambinti ir pasitarti su juo, nes be abejo, kartais pačiam žmogui jeigu ligoninis yra silpnas, jisai, va kaip sakiau tavo įtakos zonoj ir tu turi priimti tada sprendimą praktiškai už abudu. Bet galbūt tu irgi ne visada tą gali tą teisingą sprendimą priimti, tai vat tokiais atvejais, sakysim, tu galėtum pasikalbėti, pasitarti su šeimos gydytoju arba tuo gydytoju, kuris vat gydo onkologu gydytoju, kuris onkologas tavo tenai šeimos nario gydytojas ir pasitarti, taip, tai būtų labai gerai. Tai aš nežinau, aišku galbūt galėtų irgi būti kažkoks laikas, kada tu gali skambinti, žinoma, naktį tu galbūt ir neskambinsi nors, gal kartais yra ekstra atvejis, galbūt ir naktį tu galėtum paskambinti. Irgi būtų labai gerai, net tas žinoma vat slaugantis žmogus jaustųsi žymiai stipriai, kai tu žinai, turėtų kažkokią tai vat dar iš šono pagalbą. (Žmona, 45 m.)
	1b. Slaugos paslaugų neprieinamumas	Ir ir slaugos ligoninės, tai be kažkokių tokių bandymų susitarti asmeniškai, nu tai tave užrašysim į eilę ir tu toj eilėj, kol kas numirs, tai ta prasme, va ta eilė taip pajuda, kol jinai pilna absoliučiai yra slaugos ligoninė ir yra patekimas ir į tenais, kol kažkas numiršta, tada

		sekantis į tą vietą. Vat maždaug tokiu principu, tai irgi jinai yra tokia, nu net neaišku, kaip organizuoti viską. (Sesuo, 65 m.)<br><br>Tai gavosi taip, kad nežinojome, kur turėtume būti, bet kai atvykome [iš miesto ligoninės į paliatyviosios pagalbos skyrių], jie sako, kad neturi vietų. Mus išsiuntė iš klinikų čia, o jie sako, kad nėra vietos. Bet aš sakau, kad darbuotojai paskambino prieš tai. Paliatyviosios pagalbos skyrius sako, kad tai reiškia, kad toje vietoje kažkas buvo paguldytas į ligoninę. Aš sakau, žinokit, mes turime turėti vietą. (Dukra, 41 m.)<br><br>Greitoji mus nuvežė priėmimą ir kai paklausiau, ar jie turi funkcinę lovą, jie sako, kad neturi. Kambarys pilnas pacientų, mačiau, kad tai bus visai blogai. Reikėjo priimti sprendimą, ką daryti [mama turėjo būti paguldyta į ligoninę dėl plaučių uždegimo] ... Aš sakau, kad mes ją pasiimame namo. Ji negalėjo pasiversti ant šono be funkcinės lovos. (Duktė, 37 m.)<br>Kitas dalykas, jeigu jai būtų pradėtų blogėti, tai per mėnesį visko nesutvarkysi, pamenu kiek mano kolegė savo tetai visus dokumentus ir viską tvarkė. Kiek laiko reikėjo laukti kol ta pagalba buvo paskirta. Mes net nebūtumėm spėję. Mums sakė, kad galime gauti funkcinę lovą, bet kiek mums būtų kainavęs gabenimas ir kaip mes įrodytumėm kad jos reikia, nes iki to laiko močiutė buvo vaikščiojanti... (Anūkė, 48 m.)<br><br>Tai (slaugymas artimojo gyvavimo pabaigoje) yra aišku pats sudėtingiausias laikotarpis, todėl, kad na esi visiškai vienas, nes iš oficialiosios medicinos nebuvo jokios pagalbos nei psichologinės nei socialinės, jokios absoliučiai. (...) Tai nebuvo taip, kad Jums bus sunku, jeigu jums reikalinga dėl vaistų nuskausminamųjų, kreipkitės vat į šitą ar šitą specialistą. Jeigu Jus dirbate, acha jus dirbate, tai gal jums reikėtų, kad pabūtų su Jumis, tai jus kreipkitės ten, Jums padės surasti priežiūra. Tai ar ar ar sakykime, ten gal jus norėtumėt, kad su ligonių pasikalbėtų ar psichologas ar, ar, ar dvasininkas irgi tokių klasuimų ir pasiūlymų nebuvo visiškai . (Žmona, 55 m.)

		Nu kas dalyvao ? Dieve, Jūs tiesiog neįsivaizduojat. Niekas nedalyvauja. Kaip nori, taip ir adryk, iš esmės. Grįžus aš visai nežinojau ką daryti. Visai. Nei kaip ką daryti. Aš labai verkiau. Vaistus reikai leisti – nežianuanei ką kviesti. Dar pirmas deinas atėjo sesutė, paskui supratau, kad nelabai gali.. Nežianu, negalvojau, tiesiog pradėjau ieškoti žmogaus, kas leistų vaistus, spaudimą matuotų. Suardau, bet, kaina, buvo baisi. Mūsų pensija ne tokai didelė. (Sesuo, 71 m.)
	1c. Paiento, šeimos ir draugų negatyvus valstybės teikaimų slaugos paslaugų vertinimas	O institucijose tai ką – tik vaistus migdomuosius suleidžia, kad nieko neprašytų. (Duktė, 45 m.)<br><br>Sakykim, labai valdiškai [elgiasi su pacientais]. Nu taip...Tiek, kiek reikia, be jokios empatijos, be nieko. Sakykim, atėjo ir ant spintelės padėjo maistą. Praėjo kažkiek laiko, pasiėmė – „tai nevalgei". O jis pats nepavalgo, jam reikia padėti. Aš supratau, kad čia jau ne pirmą kartą [vadino negražiais žodžiais]. Mama puolė manęs atsiprašinėti, kad taip atsitiko <> Kai į ją taip kreipėsi, tai mamai tiesiog kirto. Aš pati pasimečiau, ėmiau drebėti, nežinojau ką daryti. Aš tik paskambinau broliui, nežinojau ką daryti, norėjau pasitarti. Bet nuo to mes jos vienos nebepalikom. Visąlaik pakaitom budėjom – vieną naktį tėtis, vieną aš ir vieną įbrolis. (Dukra, 37 m.<br><br>Bet jinai numirė toj ligoninėj, kai mes, liko vos ne toks, kaip jos protestas ir buvo tas, jinai tikrai numirė dėl fiziologinių dalykų, dėl tos ligos ir taip toliau, bet toks vaizdas, kad tiesiog žmogus ant tiek nenorėjo, kad ta mirtis tiesiog išsprendė tą visą reikalą. 9 Marti ,56 m.)<br><br>O institucijose tai ką – tik vaistus migdomuosius suleidžia, kad nieko neprašytų. (Vyras, 65 m.)<br><br>Be tuščios kišenės neik. Du kartus nueini, trečią kartą, kažkaip kažko reikia, kad atkreiptų dėmesį, kad atkreiptų dėmesį, kad ten vaistus parinktų, pasidomėtų. (Sesuo, 71 m.)

		Mes ją paėmėme iš ligoninės labai blogos būklės ... Aš negalėjau pažinti močiutės, ji rėkė, keikėsi, aš nežinau, kas jai buvo. Visi ten stebėjosi, kaip mes ją parvežame namo, nes mums bus labai sunku slaugyti. Ji kalba garsiai, šaukia ... Tikriausiai dėl kažkokių vaistų. Kai grįžome namo, viskas susitvarkė, ji nusiramino. (Anūkė, 49 m.)<br><br>Bet ji [serganti mama] buvo pakraupusi nuo to, kad dienos centre, kur daro chemiją, nėra vietų ir lovos koridoriuose, neatitinka poreikių. Sako, siaubas kažkoks, mes ne kaip žmonės. Tiesiog netelpa žmonės.(Dukra, 40 m.)<br><br>Nes slaugoj, slaugoj yra nustatyta du kartus keisti pampersus ar tu dažniau tuštinsies ar tai rečiau. Vienu žodžiu, jeigu rytą 9.30 ir vakare 17.30, ir tu gali apsidėti iki ausų ir tavęs niekas neateis dažniau, nepakeis ir arba tu pats turi sėdėt slaugoj. Ką jau slaugoj sėdėt, geriau jau namuose tada. Nes namai vis tiek yra namai. (Vyras, 65 m.)
**2. Artimųjų moraliniai įsipareigojimais ir prisiimtos atsakomybės**	2a. daryti teisingus dalykus	Mes ant tiek buvom surištos viena su kita, kad nu kažkaip net natūraliai atrodė, kad tik aš galiu tą daryti, Ir klabai siauros pasirinkimų galimybės, labai, nu kad akivaizdu, kad niekam kitam neišeina. (Dukra, 45 m.)<br><br>Šiaip tai tik mūsų abiejų reikalas. (Dukra, 43 m.)<br><br>Kažkaip namuose mes visi tai į vieną pusę, į vieną kryptį kalbėjom, nu kad reikia į ligoninę. Bet va, su ja pačia buvo tikrai labai sudėtinga. (....) (...) Kada ji gulėjo ligoninėj, kada liga buvo tiek pažengusi, kad namuose jau nebegalima, bet jinai sąmoningai, jinai nebuvo psichiškai neadekvačiai besielgianti ir nepasirašė į slaugos ligoninę, jinai nesutiko nei už ką. (Marti, 56 m.)
	2b. Artimųjų ir draugų neturėjimas	Aš iš esmės pats rūpinausi savo tėčiu, kasdieną važiuodavau pas juos. Mama po operacijos tuo metu buvo silpna. Aš nupirkau butą virš jų savo vyresnei dukrai, tikėdamasi, kad ji padės jai rūpintis tėvais, bet ji mokėsi ... Jai buvo sunku. <> mano sesuo atvyko iš JAV likus dviem savaitėms iki jo mirties ... (Sūnus, 48 m.)

		Iš tikrųjų viskas buvo ant mano pečių. Brolis su negalia, mama nepajėgė ... Ji tapo kitu tuo metu mano slaugomu asmeniu. (Dukra, 41 m.)<br><br>Ai, aš norėjau pasakyti tokį dalyką. Jinai gyveno su vyru, bet tas vyras prieš pat tą jos ligas insultas jį užpuolė. Tai insultas ir jinai buvo, jinai ištekėjo už našlio, jinai neturėjo vaikų ir jie užaugino du vaikučius. Vienas baigė išvažiavo į Alytų, kitas į Vilnių, dirbo banke. Ir tai reiškia tą tėvą reikia prižiūrėti, tai jie iš pradžių sako, kad aš tikrai neapsiimsiu dviejų ligonių, dėl to, kad jo charakteris buvo sunkus. Aš žinau ką reiškia, nu bijojo dviejų ligonių. Nu ir tas sūnus pasiėmė tą vyrą. Pasiėmė tą vyrą ir mes abidvi gvenom jos bute, o jis prižiūrėjo, nu tai mes susikalbindavome tik tiek. Iš jo pagalbos nebuvo. Ir iš kitų, iš kitų,<br>. Va šitoj vietoj jo, šita vieta pas mus yra, tai jau čia jo. Čia jau yra tie, ne ne nu ta prasme, kad čia yra tikrai šeimos problema. Aišku jo, jinai labai dažna, dažniausiai tas rūpinimasis pasidalinimas, bet čia ne, čia yra nerūpinimosi problema čia yra įsisenėjusi problema, kadangi tėtis labai, labai senai mirė, tikrai sūnūs, sūnūs trys labai panašaus amžiaus. Tėtis mirė, kai buvo jaunos šeimos ir jisai 50 metų mirė, tai tikrai labai jaunas. Tai va ir liko, liko ta prasme, kažkoks supratimas, kad kad mama jau viskas, jau turi išdalinti, parduoti ir eiti pas kažkurį, nu moteriai penkiasdešimt keli ten metai, tai dabar juokingai atrodo, bet kai sūnums buvo trisdešimt jiems atrodė, kad jau viskas, jau na, tai ir turtų jokių nebuvo absoliučiai, butas, mašina, garažas, nu ten juokingi tie dalykai. Bet buvo supratimas, kad mama jau turi viską išdalinti ir va pyktis atsirado nuo tada, nuo tokio, jinai tiek, jinai gyveno praktiškai, mes vat vienintelis sūnus, kuris bendravo. (Marti, 56 m.)
	2c. Slaugančiojo artimojo profesinė kvalifikacija ir turima patirtis	Mano šeima į mano rankas atidavė viską, kas susiję su jo priežiūra. Bet tas atidavimas reiškia ir atsakomybę. Jie maždaug „tu prie medicinos, tu geriausia viską žinai, tu viską tvarkai <> Aš jau pripratus. Jau seniai man ta atsakomybė. Priėmiau kaip normalų dalyką...Savaime aišku, kad tai aš. (Dukra, 47 m.)

		Iš esmės aš rūpinausi močiute. Mama buvo šalia, ją kalbindavo, bet aš padariau visą slaugą. Aš nebijau, žinau, kaip tai padaryti, jei ji vis tiek nenori to daryti, nes žinau, kad tai reikia padaryti. Tarkime, paskutinėmis dienomis visomis priemonėmis mes ją pakeliame, o mama sako, kaip jūs ją varginate. Aš sakau mama, todėl reikia, bus blogai, jei mes nesutvarkysime. (Anūkė, 48 m.)<br><br>Aš pati esu slaugytoja, vis dar dirbanti psichiatrijoje, tai nuėmiau močiutės haliucinacijas. <> Čia turime du butus. Aš nusipirkau su mintimi, kad galbūt man reikės slaugytį ją kai sirgs. Ir man reikės kambario, mes netilpsime visiems. (Anūkė, 49 m.)
	2d. Emociniai slaugančiojo artimojo ir sergančio šeimos nario ryšiai	Su tėvu palaikiau labai gerus santykius, su juo bendravome meiliai, nors jis garsiai nepasakė, kad „aš tave myliu“. <> Jam visada rūpėjo tai, ką aš dariau. Jam dar dirbant, kartą per savaitę susitikdavome mieste papietauti. Buvo toks ryšys. Kiekvieną sekmadienį eidavau pas savo tėvus su savo vaikais. Tokio ryšio su mama nebuvo. (Sūnus, 48 m.)<br><br>Kažkaip namuose mes visi tai į vieną pusę, į vieną kryptį kalbėjom, nu kad reikia į ligoninę. Bet va, su ja pačia buvo tikrai labai sudėtinga. (....) (...) Kada ji gulėjo ligoninėj, kada liga buvo tiek pažengusi, kad namuose jau nebegalima, bet jinai sąmoningai, jinai nebuvo psichiškai neadekvačiai besielgianti ir nepasirašė į slaugos ligoninę, jinai nesutiko nei už ką. Na ir ką, negalėjom kitaio.. (Dukra, 57 m.)<br><br>Manau, kad svarbiausia yra tai, kad mes buvome šalia visą laiką. Mes jos nepalikome. Nuo ligos pradžios - kuo daugiau visi kartu. Jei ne vienas, tai kitas. Ir ligoninė, mes tiesiog gyvenome. Visą parą. ... gal tai buvo ryšys tarp mūsų ... Aš buvau ramesnė sau, kai pati buvau su ja. Net kai buvo mano brolis, aš vis skambinau ir klausiau, kaip ji. (Dukra, 52 m.)<br><br>Jam tuo metu buvo svarbiausia nebūti vienam ir kad artimas žmogus su juo būtų, nes jis man yra pasakęs anskščiau, kad niekas manęs geriau neprižiūrės, kaip tu. Tai aš taip ir

		supratau, kad taip ir turi būti, kad aš esu tas žmogus, nes ir, ir, ir, ir mirties proceso metu yra svarbu, kad kažkas yra, kad tu nesi vienišas. (Žmona, 55 m.)
	2e. Sergančiojo draugų ir artimųjų slaugos ir bendravimo įgūdžių trūkumas	Bet dažniausiai vis tiek lieka, tiktai tas vienas žmogus, kuris slaugo, pagrindinis. Nes neis ten išeilės giminė slaugyti, nes per daug tada sudėtinga - ir įpročius reikia žinoti.. Tai pagrinde tada lieka vienas žmogus. Ir net tas sergantysis tada pripranta prie to vieno žmogaus. Jis nelabai nori kitų žmonių matyti. Tai kad ir su tuo pirmu vyru, jis net neprisileido kitų žmonių, kad jį slaugytų. Tai va, tai vis tiek finale iš savo patirties aš darau tokią išvadą - lieka tik tas vienas žmogus, kuris slaugo. Pagrindinis lieka, visi kiti tai ateina, aplanko, išeina.(Žmona, 57 m.)<br><br>Žmonės nėra mokomi būti su kito žmogaus kančia. Tai vat. (Dukra, 55 m.)<br><br>Ir artimi žmonės, kurie anksčiau bendraudavo su juo dabar atsitraukė ir tik paskui aš supratau, kad jie nežino ką daryti toje situacijoje, jie nežino, jie bijo skausmo, bijo prisiliesti prie skausmo, jie nežino ką kalbėti, jie tiesiog deda į krūmus liaudiškai tariant niekur. Tai va. Man ta kančia yra sava ir aš nuo jos negaliu pabėgti ir as nenoriu pabėgti. Man yra gera daryti artimam žmogui gera, taip kaip jam reikia, tai kaip aš suprantu kaip mes mums atrodo. Tai mano yra (kita) situacija. O kitiems yra rūpestėlis bereikalingas, našta, kurios jie nežino kaip pakelti, nežino ką su ją daryti. Žmonės nėra mokomi būti su kito žmogaus kančia. Tai vat. (Žmona, 55 m.)<br><br>Jinai nenorėjo su niekuo bendrauti. Jos valia, kad nieko.(...). Todėl, kad žmonės būtų atėję ir dar daugiau išmušę iš pusiausvyros ir ir ir tie tokie gailesčio žvilgsniai, gailesčio žodžiai, gailesčio judesiai tokie sakykim, kad jau visi žino, kad jau esi pasmerktas ir tu dar ateini ir dar jaudini dar vidinę pusiausvyrą. Va čia žmogus pats užsidaro .) ((Vyras, 65 m.)<br><br>Jinai turėjo tas drauges, prie draugių visą kitą, bet tos draugės kai ateidavo ją tiesiog išderindavo, pliurpia, pliurpia atsisėdę, o Viešpatie, jos kvatoja tai jos tą, tai jos tą, tai jos

		žinojo visą kitą apie savo tas. Jai to nereikėdavo, jinai sako, aš taip nuo jų pavargstu, po biški, po biški tas drauges kažkaip atsirinko, atsirinko ir pasiliko tokios dvi. Nu ateidavo tos kaimynės nu aš aš įsivaizduoju svetimam žmogui sakyti, ką gali pasakyti. Nu gali pasakyti ar tau reikia padėtį. Ne, man nereikia man sesytė yra, man to nereikia, nereikia ir viskas ir be reikalo sukuosi, nieko neišeina, dėl to, kad (dvasingas) žmogus žino kaip čia yra. Nu pasakoja visokius va tokius šiek tiek reikia, bet vot tokius. (Sesuo, 71 m.)
**3.“Tai mūsų karta: kultūriniai aspektai**		Mano vaikai manęs nežiūrės taip, kaip aš ... Mūsų kartai niekada nekilo mintis paguldyti savo tėvus į slaugos ligoninę. Nebent nutrūktų jūsų tarpusavio santykiai. Bet mano vaikams tai nebūtų jokia problema. (Duktė, 59 m.)<br><br>Aš tuo metu dar dirbau, o brolis man padėjo namuose prižiūrėti mamą. Motina vis sakydavo „nepalik savo darbo“. Aš atostogavau tris savaites, o atostogų pabaigoje pasakiau, kad pabandysiu pratęsti atostogas prašydamas nemokamų dienų. Ji kelis kartus pakartojo: „Prašau, neatsisakyk savo darbo“. (Dukra, 52 m.)<br><br>Mano vyras to jausmo [kaltės/nepatogumo, kad juo turi rūpintis] neturėjo. Jis sakydavo tu kaip šventoji, bet taip, tarsi reikia. (Žmona, 59 m.)<br><br>Net nežinau, tik kartais pagalvoju, kad mūsų vaikai į viską žiūrėtų kitaip. Ir galbūt nenorėčiau, kad jie atsisakytų darbo, studijų. Bet norėčiau, kad artimi santykiai išliktų. Na, gal ta karta ... Bet aš norėčiau, kad liktų tie artimi ryšiai. Jausti tuos žmones šalia. Tai nereiškia, kad visko atsisakytų, nors aš nesigailiu. Jų karta yra kitokia ... (Dukra, 52 m.)
**4. Slaugantysis jaučiasi atsakingas už viską**	4a. Praktinės slaugos problemos	Esu baigusi pedagogiką. Aš sakau, kad alpdavau kraują pamačiusi [juokiasi]. O čia yra daug daugiau nei kraujas. Namuose reikėjo viską daryti - perrišinėti, maišelius keisti ... mama negalėjo. Tik pirmos dvi savaitės buvo labai sunkios. (Dukra, 41 m.)

		Aš pati išmokau daryti intraveninę infuziją namuose. Kiekvieną dieną ir naktį dariau tuos užpilus. ... Vienintelis dalykas, kurio nežinojau, buvo tai, kaip įdėti šlapimo kateterį. (Žmona, 46 m.)<br><br>Kai parsivežiau ją iš ligoninės namo, paguldžiau į savo lovą, bet pamačiau didelį jos skausmą. Taigi, internete radau skelbimą apie funkcinę lovą, specialų čiužinį ir išsinuomojau visus reikalingus daiktus. (Duktė, 37 m.)<br><br>Pati išmokau lašelines namuose statyti. Kasdien dieną ir naktį tas lašelines stačiau, maitinau baltymais . Viską išmokau daryti pati, tik kateterio įdėti nemokėjau. (Sesuo, 73 m.)<br><br>Išleido namo ir ir nu kaip namuose, aš atsimenu tik labai baisų dalyką, kada atvažiuoji ir galvoji Viešpatie, kad kas nors parodytų kaip čia daryt kaip ką. Nu tai, tada aš pasiskambinu jos gydytojai aš buvau pas ją nuėjus šeimos gydytoją. Sakau daktare, duokit moterį, kuri man parodytų ir kad aš drebančiom rankom, kad žinočiau ką daryti. Jeigu ten suskaudo, jeigu ten paraudo, kad kas nors ateitų, kad ir patronuojanti sesytė. Nu tai gerai, nu tai gerai, bet žinot, kaip pas mus žmones jų nėra ir visą kitą. Tai sesytė, ateina sesytė apsižiūri 10 eurų, 10 eurų keturiolitais metais, eurai buvo ar ne? (Sesuo, 71 m.)
	4b. Konsultavimas ir informavimas	Hematologas pirmiausia mane pakvietė ir pasakė diagnozę. Ir paklausė, ar jis galėtų apie tai kalbėti su mano vyru. (Žmona, 46 m.)<br><br>Onkologas manęs paklausė, kokį gydymą pasirinksime: tradicinį ar alternatyvų. Aš žinojau, ką reiškia tradicinis, jie paaiškino, bet aš nebuvau tikra dėl alternatyvaus ... todėl ieškojau kontakto, žmonių, kurie galbūt žino daugiau. (Žmona, 46 m.)<br><br>Skambindavau gydytojai onkologei ir klausdavau, ar turėčiau jai pasiūlyti maisto, kai ji atsisako ... Kartais labai supykdavau, nes mama atsisakė vartoti vaistus, aš nežinojau, ką daryti. Aš paklausiau jos gydytojos, kaip turėčiau pasikalbėti su mama, kad įtikinčiau ją gerti

		tuos vaistu. ... Ir ką daryti skausmo atveju. Kokie vaistai padėtų? Žinote, viskas nauja, nežinai, ką daryti. (Dukra, 52 m.)<br><br>Mes nežinojome, ar jis [vyras] suprato savo sveikatos būklę. Nenorėjau paklausti, ar jis žino, kas vyksta. O jei jis paklaus: „kas su manimi vyksta? Ką pasakyti? Toliau meluoti? (Žmona, 68 m.)<br><br>Man ne reikėdavo stovėti prie durų ir maldauti, kad tu mane priimki, nes baigiasi vaistai nuskausminamieji, o ten daugumai reikia be eilės, nes negali išsirašyti į priekį kiek tau na užsirašyti iš anksto negali ir, ir, ir tu nežinai, kada tau prireiks dar daugiau tų vaistų, nes dozes jau reikės keisti, stiprinti, tai va. (Žmona, 55 m.)<br><br>Viskas vyko tariantis su gydytoja. Kai norėjom, kad perkeltų į kitą, kaip mums atrodė, geresnį skyrių, tai dar tarėmės ir su Birute, ir su pussesere. O dėl gydymo – buvo, taip, reikėjo priimti sprendimus. Rimtus. Jai galima buvo įvesti kažkokį vamzdelį į plaučius, kad palengvintų kvėpavimą. Tada gydytoja su mumis tarėsi, mes su vyru turėjome priimti sprendimą. (Marti, 55 m.)
	4c. Vadybinimas	Aš skambindavausi su jos šeimos gydytoja, su slaugos ligoninės gydytoja pasitarti. Tuo metu buvo karantinas, į slaugos ligonines nieko nepriiminėdavo, ir jeigu priimtų nieko daugiau. <> Mes kalbėjome su šeimos gydytoja, ar reikia perrašyti arčiau, į tą miestelį, kur močiutė persikėlė gyventi. ..(Anūkė, 48 m.)<br><br>Ačiū Dievui, mes turėjome kaimynę, kuris buvo slaugytoja, dabar pensininkė. Paprašiau jos atvykti į suleisti vaistų į veną, kai mama sirgo plaučių uždegimu. (Duktė, 37 m.)<br><br>Aš eidavau pas šeimos gydytoją, jos šeimos gydytoja kuri jautė gal kad pražiūrėjo biški, pavėlavo visą kitą. Jeigu kas aš jau pasiskambindavau ir ir buvo tokia patronuojanti sesytė prie gydytojos sesytė tokia (...) kuri pas mus ateidavo nuo tos daktarės, daktarė klausia taip, daktarė klausia taip, kokių klausimų turit. Nu kaip aš sakau, penki eurai į kišenę ir jinai ateina,

		niekas neturėdavo laiko. Gydytojo konsultacija tokia medicininė būdavo gal ko reikia, gal to reikia. Paskui vieną kartą, koją jai labai traukė, koja, kojos nebepriminė aš nulėkiau pas tą daktare sakau, ką dayti, ką reikia daryti, tai jinai, paskui važiavom pas tokį, ten baksoja tą koją, pas tą kažkokį prirašė, dar paskui prirašė daryti fizioterapeutą man atrodo, kad jai lankstino pirštus, padus tuos ir visą kitą ir čia šlaunis. (Sesuo, 71 m.)
	4d. Būti kartu: emocinis sergančiojo artimojo palaikymas	Tėvas po chemoterapijos beveik apako. Jis negalėjo nieko daryti, išskyrus klausytis radijo ir televizijos. Jis norėjo bendrauti <> Jis tiesiog norėjo, kad kažkas būtų šalia, kalbėti. Net kalbėti kartais nenorėjo .... Jis tiesiog norėjo matyti savo šeimos narius. Na, žinot, kad jis neužmirštas ... (Sūnus, 24 m.)<br><br>Aš rūpinausi juo kaip zombis. Jis privertė mane tiek kentėti per mūsų gyvenimą kartu. ... Bet aš padariau visus reikalingus dalykus. (Žmona, 68 m.)<br><br>Jam tuo metu buvo svarbiausia nebūti vienam ir kad artimas žmogus su juo būtų, nes jis man yra pasakęs anskščiau, kad niekas manęs geriau neprižiūrės, kaip tu. Tai aš taip ir supratau, kad taip ir turi būti, kad aš esu tas žmogus, nes ir, ir, ir, ir mirties proceso metu yra svarbu, kad kažkas yra, kad tu nesi vienišas. (Žmona, 57 m.)<br><br>. Buvo turbūt svarbiausia šeima ir palaikymas. Manau, jisai mūsų turėjo sūnų, sūnus jam buvo labai svarbus, anūkas buvo svarbus nu be abejo ir aš buvau labai svarbi. Ir jam buvo svarbu, kad šeima būtų šalia, kuo daugiau šeima būtų šalia. (Žmona, 51 m.)
	4e. Tu privalai pasilikti čia; negatyvios buvimo ligoninėje patirtys	Man buvo pasakyta „kodėl tu ten [ligoninėje], juk ligoninėje yra slauga ir priežiūra. Iš savo patirties pasakysiu, kad jūs turite būti šalia. Žinoma, jūs pavargęs .... Kai esate ten, jie [darbuotojai] rūpinasi juo labiau. Aš mačiau ir supratau, kad jūs turite ten būti, turite prisitaikyti <> vakarais trūksta darbuotojų ir reikia artimųjų pagalbos. (Dukra, 41 m.)

		Na, mačiau, kaip jos [slaugytojos] elgėsi su pacientais ... su tais, kurie neturi kas juos lanko palatoje. Kaip pasakyti ... labai formaliai. Pavyzdžiui, atneša maisto, padeda jį ant spintelės šalia lovos ir palieka. Kurį laiką jie vėl ateina, sako: „Tu nieko nevalgei“ ir išneša. Bet tokioje situacijoje esantis pacientas negali pats valgyti, jam reikia kažkieno pagalbos. (Sūnus, 48 m.)<br><br>Aš supratau, kad čia jau ne pirmą kartą [vadino negražiais žodžiais]. Mama puolė manęs atsiprašinėti, kad taip atsitiko <> Kai į ją taip kreipėsi, tai mamai tiesiog kirto. Aš pati pasimečiau, ėmiau drebėti, nežinojau ką daryti. Aš tik paskambinau broliui, nežinojau ką daryti, norėjau pasitarti. Bet nuo to mes jos vienos nebepalikom. Visąlaik pakaitom budėjom – vieną naktį tėtis, vieną aš ir vieną įbrolį. (Dukra, 50 m.)<br><br>Galbūt [pinigų] davimas ar nedavimas nieko nepakeis, bet manai, kad kažkas bus geriau. Daug apie tai negalvoji. Nors chemoterapijoje mes nedavėme pinigų, tik kokią dovaną <> net nemanai, kad tai kyšis - toks malonus ženklas. (Dukra, 52 m.)
	4f. Finis ir emocinis išsekimas	Mano mama bijojo likti viena namuose. Taigi psichologiškai darėsi vis sunkiau. Ypač naktį. Ji paprašė manęs ateiti ir būti su ja. Negalėjau normaliai miegoti ... Jaučiau, kad išprotėsiu... 24 valandas namuose su ja .... (Duktė, 37 m.)<br><br>Ir dar vienas labai svarbus dalykas. Jei žmogus tiki Dievu ... Kai buvau labai pavargusi, grįždavau namo, mamai reikėjo pagalbos, brolis buvo įsitempęs, nes tėvas buvo geriausias jo draugas ... Apskritai ... vis tiek turi atlaikyti visus tris. Gerai, kad aš tikiu. Aš labai prašiau kitų maldų. Mano sveikata buvo tokia ant ribos ... (Duktė, 41 m.)<br><br>Vis ko nors reikėdavo atsisakyti. Pavyzdžiui, nu, daugiausia tai susieta su savo irgi gyvenimu ir savo laisvalaikiu kažkokiu savo norų patenkinimu. Kartais tiesiog, kadangi, tiesiog, kol neišeidavau į darbą, tai visą laiką būdavau kartu. Iš pradžių dažnai kartais norėdavosi bėgti ir išbėgdavau, palikdavau, paskui grįždavau ir šitą ir mano draugė sakydavo, kaip man baisu,

		kai mane palieka ir aš žinau, kad aš pati nieko negaliu. Paskui stengdavausi nebėgti, kai būdavo sunku atsiguldavau ten, melsdavausi. (Dukra, 45 m.)<br><br>Man buvo sunku ir nu tai čia irgi galbūt aš kažkokios šiek tiek tokios sąžinės priekaišto jaučiu, kad aš pasakydavau, oi kaip aš pavargau, nes aš jau tiesiog labai pavargusi buvau, kad nu nesulaikydavau....tai gal ir nelabai aš čia su tuo orumu ...jo. (Marti, 55 m.)
	4g. Pasekmių įveikimas	Nu labai nesinorėjo išduoti, nors kartais atrodė, kad jau vidus tai ir galėtų ten kažką padaryti ir įskaudinti, va. (Žmona, 71 m.)<br><br>Vis ko nors reikėdavo atsisakyti. Pavyzdžiui, nu, daugiausia tai susieta su savo irgi gyvenimu ir savo laisvalaikiu kažkokiu savo norų patenkinimu. Kartais tiesiog, kadangi, tiesiog, kol neišeidavau į darbą, tai visą laiką būdavau kartu. Iš pradžių dažnai kartais norėdavosi bėgti ir išbėgdavau, palikdavau, paskui grįždavau ir šitą ir mano draugė sakydavo, kaip man baisu, kai mane palieka ir aš žinau, kad aš pati nieko negaliu. Paskui stengdavausi nebėgti, kai būdavo sunku atsiguldavau ten, melsdavausi. (Dukra, 45 m.)<br><br>Man buvo sunku ir nu tai čia irgi galbūt aš kažkokios šiek tiek tokios sąžinės priekaišto jaučiu, kad aš pasakydavau, oi kaip aš pavargau, nes aš jau tiesiog labai pavargusi buvau, kad nu nesulaikydavau....tai gal ir nelabai aš čia su tuo orumu ...jo. (Žmona, 61 m.)<br><br>Tai esi su tuo ligonių vienas ir kažkaip bandai kapstytis, ir tas toksai beviltiškumas ir toks fizinis ir protinis išsekimas vien dėlto, kad tu negali pailsėti visiškai, tu turi daryti ir kas namuose reikalinga slaugant ligonį ir eiti į darbą ir ir ir dirbti darbo dieną. (Žmona, 55 m.)<br><br>Dažnai labai pykdavausi šiuo paskutiniu jo gyvenimo tarpsniu, dėl ko dabar save kaltinu. Jis buvo labai ramus. Namie jis buvo taikdarys. <> Kai jam praeidavo pykčio priepuoliai jis atsiprašinėdavo. O aš jo neesu atsiprašiusi… (Žmona, 47 m.)
