# Supplementary material for: What does it mean to be the main caregiver to a terminally ill family member in Lithuania?: A qualitative study
Source: PLoS One. 2022 May 12;17(5):e0265165. doi: 10.1371/journal.pone.0265165 (PMC9098011; doi:10.1371/journal.pone.0265165)
Supplement: S5 File — (DOCX) [file pone.0265165.s005.docx]

**Supplementary File 3.**

**Samples of original Lithuanian interview texts.**

Full transcripts are available.

**I –** interviewer

**P –** participant

**Moteris, 55 m., slaugė vyrą**

**I.***: O kaip pati galvoji, jeigu nuo pat pradžių būtų buvusi tokia visapusiška pagalba. Tai, kaip matai ar būtų tas paskutinis gyvenimo laikotarpis buvęs kažkoks kitoks?*

**P**: [...] Manau, taip. Manau, žiniai kitoks, todėl, kad man reikėtų tada, kaip sakyt, daug veiklos daryti be tikslės. Nekalbu apie tai, kad reikėjo specialius poreikius ten organizuotis, lakstyti po po po institucijas, popierius nešioti, laukti eilėse. Nepasinaudojau aš tais poreikiais, kol popieriai susivaikščiojo, žmogus mirė jau. Ir poreikiai buvo penkiasdešimt ar ne šeši eurai nustatyti. Ką už 56 eurus, kokius poreikius tu gali žmogaus tenkinti, net sauskelnes poreikių negali tenkinti, nieko, tai, tai. O paskui matyt tada kada būtų jau buvusi, ta jaukita,nes yra laikas nustatyta tam tikras laikotarpis per kiek laiko žmogui gali pablogėti, jam negali pablogėti per dieną per ar dvi, matyt per mėnesį per du. Vienu žodžiu, tokie kaip sakyt, dėmesio žmogui nėra jokio nei tam pacientui nei slaugytojui. Yra kažkur kažkokie aktai teisės, bet jie yra kažkur danguj, prie to konkretaus žmogaus kuriam jų reikia, jie neatsiranda, jie pats turi ieškoti, o kad ieškoti, dar turi žinot kur ieškoti, tai va. Tai aš manau, jeigu tik atsiranda tokia diagnozė žmogui nustatoma, jis turi gauti visapusišką informaciją kur ir kokių klausimų jis turėtų kreiptis. Tada galima ta laiką planuoti taip, kaip galima geriausiai ir pacientui ir pačiam slaugytojui, nes jei slaugytojui blogai, tai ir pacientui blogai

.............................................................

**Moteris, 51 m., slaugė vyrą**

**I**: *O kaip slauga vyko?*

**P***: ...* Jisai muzikos klausydavosi, jisai, kai vienas būdavo tai daug klausydavosi, turbūt ir melsdavosi taip ir miegodavo na taip jau. Kažkokio komforto didelio na nežinau ar ar ar ar ar yra galimybė suteikti. Lova ta yra, be tos funkcinės lovos būtų neįmanoma, nes aš pati asmeniškai aš negaliu daryti fizinio darbo, negaliu nei pasilenkti, nei kelti sunkai. Tai be funkcinės lovos yra tada viskas. Reikia arba kažką pasamdyti, arba atiduoti į kažkokią ligoninę. Funkcinę lovą samdžiau, pati nuomavau, niekas man nepasakė, kad tu gali, tau aš išrašysiu, tu gali nuomuotis, skolintis, kad yra tarnybos, kurios skolina tuos dalykus visus. Informacijos jokios apie tai. Tai aš pati reiškia susirandu internete ir tada bėgu ir nu ieškau, tai ir deguonies aparatą pati nuomavausi ir ir tą funkcinę lovą ir genetinai brangiai. Paskui tik tai, kaip mamai reikėjo slaugyti atradau, kad vos ne per pus pigiau galima buvo nuomotis. Bet irgi nuomavau pati irgi nežinojai, jokios tokios vat socialinės kažkokios techninės pagalbos informacijos jokios neturėjau. Tai dar vat tokių papildomų lėšų išsileido, kur galbūt galėjai ir neturėti jų.

......................................................................................................................

**Moteris, , 54 m., slaugė anytą**

**I:***. Ką Jums reiškia rūpintis sunkiai sergančiu artimiuoju? Kokios Jūsų patirtys??*

**P**: Bet iš tikrųjų, kur dėti tokį žmogų, kuris, nu tikrai negali tu jo, nes mes irgi dirbam ir negali tu jo pasivežti, tu neišsprendi tos problemos, nes tu grįžti vakare ir ir ir kas visą dieną ją rūpinsis. Ir tas nerimas buvo jau seniai, kad ar jinai ir vaikščiojo ir iki parduotuvės nueidavo ir viską, bet jau matėm, kad viskas artėja, kad tikrai kažkada viskas bus, taip, kad reikia ir tu visai nežinai kur kreptis, ką daryti, kaip išspręsti tą dalyką.

.....................................................................................................................

**Moteris, 69 m., slaugė seserį**

**I:** *O kokios Jūsų patirtys? Kas buvo sunkisia slaugant seserĮ*

**P**: Išleido namo ir ir nu kaip namuose, aš atsimenu tik labai baisų dalyką, kada atvažiuoji ir galvoji Viešpatie, kad kas nors parodytų kaip čia daryt kaip ką. Nu kur rasti kokią informaciją? Nu tai, tada aš pasiskambinu jos gydytojai aš buvau pas ją nuėjus šeimos gydytoją. Sakau daktare, duokit moterį, kuri man parodytų ir kad aš drebančiom rankom, kad žinočiau ką daryti. Ir bendrai, kaip man ja rūpintis? Kas gali padėti? Nu juk jokios, jokios informacijos...

.....................................................................................................................

**Dukra, 36 m., slaugiusi mamą**

**I**: Ema, ar tau kažkas padėjo, ateidavo pakonsultuoti?

**P:** Niekas. Paskambinau į maltiečius, sako gal po pusės metų atsiras galimybė. Šeimos daktarei tai kyšius mokėdavau, kad bent ateitų. Toks tarybinis kažkoks mąstymas. Kad nors ateitų. Kai sirgo plaučių uždegimu, tos venos labai prastos. Tai gyveno kaimynė sesutė pensininkė, jos paprašydavau, ji ateidavo ir mes bendrom jėgom tuos antibiotikus. Bet kad ateit, suleist vaistu...

**I:** Niekas nepasiūlė?

**P:** Nieko nepasiūlė, kiekvienąkart su kyšiais. Tai ta sesutė man padėjo. Gerai, kad turėjom pinigų, tai visi tepalai, pampersai...

**I:** O gydytoja neparašė dėl pampersų?

**P**: Apie tai sužinojau visai netyčia kai pirkau ėjau paiimti fentanilio ir man vaistininkė pasakė. Tada pyktelėjau.

**I:** Tada pasakėt gydytojai?

**P:** Pasakiau, tai ...Toks vaizdas, kad ji taupo.

....................................................................................................................

**I:** Ko trūko tau mamos priežiūros procese kalbant apie orią mirtį?

**P.** Pati viską dariau. Gerai, jeigų būtų atėjusi pagalba, gal socialinė darbuotoja ar kas. Sausio mėn. man buvo visai blogai ir aš sugalvojau kartu su mama, tada ji dar geriau jautėsi, kelias valandas grįžti į klinikas padirbti. Nes man nugarą vis labiau skaudėdavo, būdavo vis sunkiau vartyti. Nusprendžiau bent rytiniam tualetui rasti moterį. Ieškojom internete, dar tokią atranką darėm ir išsirinkom tokią moterį turinčia daug metų patirties. Ir ji taip mokėjo lengvai, grakščiai vartyti. Mamai patiko ta moteris. Tai džiaugiuosi tuo sprendimu, kad man visai stogas nenuvažiuotų. Taip porą mėnesių iki mirties.

I: Tokios praktinės pagalbos?

**P:** Taip, galų gale ir plaučių uždegimu sergant reiktų vaistus suleist. Man pačiai stresas, dar daktarė pasakė, kad greičiausiai neišgyvens, tai man dar didesnis stresas.

**I**: Sakai, kad į namus socialinė darbuotoja. Kokios pagalbos iš jos?

**P:** Nežinau, bandau lyginti su Anglija. Ta socialinė ten viską tvarkė. Pradedant nuo reikalingų priemonių iki to, kad surado slaugos ligoninę, domėjosi kaip sekasi, siūlė hospisą, slaugos ligoninę, dėl nuskausminamųjų, ji skausmą ištirdavo. Aš jaučiausi saugi ir ne viena. O Lietuvoj buvau viena.
