# Supplementary material for: What does it mean to be the main caregiver to a terminally ill family member in Lithuania?: A qualitative study
Source: PLoS One. 2022 May 12;17(5):e0265165. doi: 10.1371/journal.pone.0265165 (PMC9098011; doi:10.1371/journal.pone.0265165)
Supplement: Attachment — Submitted filename: Response to reviewers.pdf [file pone.0265165.s007.pdf]

Manuel Fernandez-Alcantara, PhD
Academic Editor
PLOS ONE

RE: PONE-21-00726

Dear Dr. Fernandez-Alcantara:

We are resubmitting manuscript PONE-D-21-00726, entitled "What does it mean to be the main caregiver to a terminally ill family member in Lithuania: the caregivers' perspective."

We have addressed the points raised by the reviewers. We have also ensured that we have adopted the PLOS ONE format, as you noted. The reviewers' comments are listed below with responses to each point. The responses are in blue text after each comment.

The new, edited text is included, along with a copy of the original text that shows highlighted changes. On this original text, the deleted material is in red, and new material is in blue.

Please inform us if additional information is required.

Sincerely,

Jolanta K

## Reviewer #1

Areas requiring attention to strengthen the submission:

1) The authors do reference some family caregiver literature in their paper, but the manuscript lacks a critical synthesis of the large body of family caregiver research that has been done to date. I would have expected to see included the seminal works of Dr. Linda Kristjanson, for example. The authors do situate their findings vis a vis some extant work, but their findings are not that surprising, and it begs the question as to why they conducted the particular study that they did. I appreciate that their might be cultural variations, but the exact gap that the project purported to fill is not compelling.

Response locations in the manuscript

a) RE caregiver research literature

Introduction: fourth paragraph, including notes on Kristjanson’s work beyond what was cited in the original manuscript.

b) RE why the study was conducted
**Introduction**: third paragraph concerning the value of Lithuania as a study site.
**Introduction**: The second, third, and fourth paragraphs further explicate the significance of direct caregiver accounts of their lived experience in a setting in which there is little or no teamwork and the illusion of services is prominent. The Conclusion now highlights this point.

c) **Introduction:** New material in paragraph two develops the nature and rationale of the Lithuanian setting.

2) To their credit, the authors identify role theory, particularly the thinking of Merton, as informing their work. The paper would have been stronger had they provided more information about the theory and its underlying assumptions, as well as a critique of its limitations. For example, not all family members will agree to undertake the caregiver role, and such a stance would be seen as deviant behavior. How does role theory account for this? To what extent does the use of role theory as a theoretical lens reinforce commonly held notions about how people should behave? This is particularly relevant given that the majority of caregivers in the study were women of a certain generation.

RE role theory
**Introduction**: The contextual use of role theory in studies of caregiving is added in the sixth paragraph, beginning “Much of the existing body of research….” The current study is part of this ongoing convention. A full explication of role theory in this inductively structured study was seen as unnecessary. Additional gender-role related work is being carried out as part of the larger project. Thus, as in most other studies of caregivers, role theory is not fully explicated in this inductive study, which uses role as a necessary starting point, offering some conceptual grounding., but not the limitations of hypotheses that would be used in very specific aspects of the phenomenon being studied.

3) The biggest limitation in the paper is that the authors invoke the notion of dignity, and dignified care, but never define the

construct, or integrate the well developed body of literature about dignity and end of life care in the paper. This is a significant gap.

RE dignity

**Introduction**. The second paragraph clarifies a working definition of dignity and places the study in the context of the larger project on dignity at the end-of-life. While dignity is an overarching concern, in this research data on dignity was found to be inductively rather than directly sought or hypothesized, and dignity was interwoven with the specific experiences of the participants.

4) The issue of corruption in paying to help ensure better care provision in institutions and/or to "move patients up in the queue is an important, albeit disturbing finding. The manuscript would have been strengthened had this finding been situated within literature that exists talking about the magnitude of the problem of corruption, and the responsibilities of researchers in all countries in helping to address it. (e.g. see Lancet 2019 https://www.thelancet.com/journals/lancet/article/PIIS0140-6736(19)32527-9/fulltext)

RE: corruption

a) The matter of corruption was further developed in in theme 4e. It is contextualized more in paragraph four of **Discussion** by noting the worldwide problem as presented by Garcia, as well as discussion of the cultural complexity of informal payments and gifts in the health care sector that have occurred in Lithuania.
b)

5) I appreciate the need to move to virtual interviews for data collection amidst the strain of the pandemic! Despite advantages including convenience and interactivity of collecting data virtually, the literature does document a number of ethical, practical, and interactional issues associated with the use of digital technologies. The paper would have been stronger had these been mentioned in the paper, and the authors' experiences compared with them.

RE: use of audio-visual media

a) **Sample**. The third paragraph adds material on the use of these media in this study and consideration of the relationship between media and data. New Table 2. (Profile of caregivers) shows breakdown of audio-visual and in-person interviews and descriptive sample characteristics.

Reviewer #2: The topic itself on “What does it mean to be the main caregiver to a terminally ill family member in Lithuania: the caregivers’ perspectives” has the potential to become an issue of caregivers. The study conceptualized caregivers’ understanding of the functions that they assume while being the main caregivers, and to understand how they experienced the consequences they confronted for filling the research gap. The paper deals with an important topic that is likely to be of interest to the researchers. In its current form, I may suggest the author modified the errors of details and enhance the final manuscript.

Introduction

(1)It is better to state more clearly about the objectives and research questions.

The Introduction includes new clarifying content.

Materials and Methods

(1)Consider the guide for the presentation of qualitative studies (COREQ)

COREQ guidance has been noted and highlighted specifically in several instances as further noted below.

(2)It is better to state clearly why 33 caregivers included in the current study?

The second paragraph in the SAMPLE section includes new material on the rationale for 33 participants.

(3)The authors adopted qualitative methods in the current study. It is better to state clearly the logic of qualitative methods. It is better to provide a table about descriptive conditions about the interviewees.

New Table 1. Presents the logic of the qualitative study.

(4)The authors should state clearly about the setting of data collection, number of participants that refused to participate or dropped out, field notes, data saturation.

New material in the Sample section addresses drop outs (COREQ #12), refusals (COREQ #13), location (COREQ #14), presence of others (CPOREQ #15). New Table 2 (Profile of caregivers) describes the sample numerically.

Results

（1） The authors should state clearly why the paper include 4 themes of the main caregiver of a terminally ill family member and the meaning of the caregiver role.

This content is enhanced in the section on Data Analysis and in the new Table 1., Logic of the study.

（2） The authors should state about number of data coders, consistency between data and findings, software used, and participant feedback.

New Table 1., Logic of the study, Phase 2 clarifies roles and numbers of data coders and verifiers. No specialized software was employed.

Discussions

(1)The discussion part should connect with the results part, at present, the themes in the part of results have not been stated in the discussion part.

The themes are now highlighted in the Discussion section. The matter of corruption was further developed in in theme 4e. It is contextualized more in paragraph four of **Discussion** by noting the worldwide problem presented by Garcia, as well as the recognition and attempts to reduce corruption in health care that have occurred in Lithuanian social policy.
