# Supplementary material for: What does it mean to be the main caregiver to a terminally ill family member in Lithuania?: A qualitative study
Source: PLoS One. 2022 May 12;17(5):e0265165. doi: 10.1371/journal.pone.0265165 (PMC9098011; doi:10.1371/journal.pone.0265165)
Supplement: Attachment — Submitted filename: Plos One.docx [file pone.0265165.s006.docx]

Thank you for the opportunity to review this manuscript.

I offer the following specific evaluative comments.

Strengths of the submission include the following:

1) Given the nascent nature of palliative care services in Lithuania, and the critical role that family caregivers play in providing care to those with life-limiting illnesses, it is important to examine and describe family caregiver experiences within this specific geographical context.
2) Overall, the paper is well organized and written.
3) The data exemplars provided help to illustrate and appear emblematic of the themes presented.
4) There is mention of a theoretical grounding for the study, i.e. role theory.
5) The sampling approaches and data analysis procedures are well described and there is some mention of the issue of rigor.

Areas requiring attention to strengthen the submission:

1) The authors do reference some family caregiver literature in their paper, but the manuscript lacks a critical synthesis of the large body of family caregiver research that has been done to date. I would have expected to see included the seminal works of Dr. Linda Kristjanson, for example. The authors do situate their findings vis a vis some extant work, but their findings are not that surprising, and it begs the question as to why they conducted the particular study that they did. I appreciate that their might be cultural variations, but the exact gap that the project purported to fill is not compelling.
2) To their credit, the authors identify role theory, particularly the thinking of Merton, as informing their work. The paper would have been stronger had they provided more information about the theory and its underlying assumptions, as well as a critique of its limitations. For example, not all family members will agree to undertake the caregiver role, and such a stance would be seen as deviant behavior. How does role theory account for this? To what extent does the use of role theory as a theoretical lens reinforce commonly held notions about how people should behave? This is particularly relevant given that the majority of caregivers in the study were women of a certain generation.
3) The biggest limitation in the paper is that the authors invoke the notion of dignity, and dignified care, but never define the construct, or integrate the well developed body of literature about dignity and end of life care in the paper. This is a significant gap.
4) The issue of corruption in paying to help ensure better care provision in institutions and/or to “move patients up in the queue is an important, albeit disturbing finding. The manuscript would have been strengthened had this finding been situated within literature that exists talking about the magnitude of the problem of corruption, and the responsibilities of researchers in all countries in helping to address it. (e.g. see Lancet 2019 <https://www.thelancet.com/journals/lancet/article/PIIS0140-6736(19)32527-9/fulltext>)
5) I appreciate the need to move to virtual interviews for data collection amidst the strain of the pandemic! Despite advantages including convenience and interactivity of collecting data virtually, the literature does document a number of ethical, practical, and interactional issues associated with the use of digital technologies. The paper would have been stronger had these been mentioned in the paper, and the authors’ experiences compared with them.
