# Supplementary material for: What does it mean to be the main caregiver to a terminally ill family member in Lithuania?: A qualitative study
Source: PLoS One. 2022 May 12;17(5):e0265165. doi: 10.1371/journal.pone.0265165 (PMC9098011; doi:10.1371/journal.pone.0265165)
Supplement: Attachment — Submitted filename: Response to Reviewers 2 June 2021.docx [file pone.0265165.s008.docx]

PONE -D-21-00726RI

2 June 2021

Manuel Fernández-Alcántara, Ph.D.
Academic Editor
PLOS ONE

Dear Dr. Fernández-Alcántara:

Thank you for your request that we address two minor points in the manuscript PONE -D-21-00726RI, previously entitled “What does it mean to be the main caregiver to a terminally ill family member in Lithuania: the caregivers’ perspectives.”

We have addressed these two minor changes as requested and consider the manuscript complete to your standards. We are attaching a file with the new, revised and complete manuscript (filename: Manuscript), and a file with the manuscript clearly showing the tracking of the new changes (filename: Revised Manuscript with Track Changes).

Please note that these files show that the following changes have been made according to your guidance:

Requested change 1. “Please modify the title of the manuscript to include the qualitative design. I will recommend something as " What does it mean to be the main caregiver to a terminally ill family member in Lithuania: a qualitative study".”

Completed change 1. We have changed the title accordingly to “What does it mean to be the main caregiver to a terminally ill family member in Lithuania?: A qualitative study”.

Requested change 2. “The first paragraph of the introduction (where authors state their objectives) should be placed at the end of the introduction (just before Material and Methods section)”

Completed change 2. We have moved the specified text to the specified place in the manuscript, just before the Material and Methods section.

We appreciate your careful reviews and guidance in preparing the manuscript for publication and look forward to final notification of its acceptance.

Sincerely,

Jolanta Kuznecovienė, PhD
