# Supplementary material for: What does it mean to be the main caregiver to a terminally ill family member in Lithuania?: A qualitative study
Source: PLoS One. 2022 May 12;17(5):e0265165. doi: 10.1371/journal.pone.0265165 (PMC9098011; doi:10.1371/journal.pone.0265165)
Supplement: Attachment — Submitted filename: Rebuttal letter_response to reviewers.docx [file pone.0265165.s009.docx]

Dear Dr. Soundy,

Regarding: POLS ONE Decision: Revision required [PONE-D-21-00726R2] – [EMID:0994672364td1bfe]

## We appreciate your comments and recommendations for revision of our study, “What does it mean to be the main caregiver to a terminally ill family member in Lithuania?: A qualitative study.” We have made changes based on the revised manuscript that was sent in response to the first reviewers’ comments. The current changes are detailed with Track Changes, and a clean version of the revised manuscript is also included.

## Here is an account of what we have done in response to each of your points.

Editor’s point 1.

“• Please make sure the reader understands the philosphical underpinnings of descriptive thematic analysis – I have had a look at your reference 27 that states: “Applied thematic analysis as we define it comprises a bit of everything—grounded theory, positivism, interpretivism, and phenomenology—synthesized into one methodological framework” – personally I find that statement extremely odd because they mention, paradigms of positivism and interpretivism which are opposites alongside methodologies of grounded theory (but don’t name a type) and phenomenology (again no type is given). You will have to reference this because a reader needs to understand how you view the world using this approach and readers will expect a statement that considers a paradigm and methodology. To me it sounds like a pragmatic approach which uses different tools from paradigms and methodologies.”

We have deleted the first sentence of the first paragraph in the Materials and Methods section, and added the following, including a new reference that will be added to the References list [**Pietkiewicz**, I., and Smith, J.A. Czasopismo Psychologiczne – Psychological Journal, 20, 1, 2014, 7-14].:

Research Strategy

The logic and procedures of the study are shown in Table 1. The research team used the strategy of applied thematic analysis as presented by Guest, et al. [27] to structure the study. Applied thematic analysis is flexible and does not propose a methodological recipe. The strategy is integrative in that it allows for the pursuit of phenomenological understanding through the use of data collection and analysis methods derived in part from a positivistic tradition. Interview text is data observed and recorded in the real world, meaning that it is positivistic data. The analysis process was designed to seek commonalities of experience reflected in this data, and to apply an interpretive, phenomenological orientation to the systematic assessment and understanding of the essential meaning conveyed by the data. The team used the interpretive phenomenological analysis method advocated by Pietkiewicz and Smith. Although Pietkiewicz and Smith did not identify the use of positivistic data for interpretive elements explicitly, they describe the use of interview (and other empirical, positivistic) data as foundational elements to construct a phenomenological presentation of how people understand a situation.

Editor’s point 2.

“• Have a separate sub-heading for the interview guide and its development”

The following new section has been added after the section entitled “Research Strategy”:

**Procedures**

**Interview Guide**

Qualitative methods of this type are particularly useful in capturing content and context that may not have been known or hypothesized a priori [27]. To find themes the team of six public health faculty members designed an interview guide that provided preliminary structure for data collection. The guide built upon the content of previous studies of caregivers’ behavior, many of which were framed explicitly or tacitly by the concept of social role. For example, Stajduhar et al. reviewed dozens of studies that emphasize the knowledge and skills of the caregiving role and how caregivers acquire them [11]. The intent of the guide was to explore the experiences of caregivers in fulfilling the caregiver role, including role elements concerning everyday life of caregiving, the needs of the terminally ill person and the family, patterns of communication and decision-making with professionals, and caregivers’ perceptions of a dignified end-of-life experience. Team members were sensitive to the changes that one might experience in the role over time. Interviewers used probes to ensure that the data were characterized by clarity, sensitivity to difficult topics, openness to important new topics, and explanations of inconsistencies [28].

Discussion to refine the early drafts of the guide and informal pre-tests led to the decision to reduce the number of stimulus questions and to frame them to allow for a broader range of responses. After analysis of the first interviews, the researchers supplemented the guide with emerging questions on topics about knowledge and use of social services and other formal and informal social supports, the aversion to and fear of using any sort of inpatient palliative care facilities, and the necessity to provide unofficial payments to medical personnel in order to receive any services. Consistent with the design of the study, these topics reflect specific Lithuanian realities that may be clearer in the country’s specific national context than they would be in different settings where theyare less apparent to practitioners, but still present. The questions stimulating by far the most valuable data were “How do you personally understand dignity at the end-of-life?” and “What factors ensure dignity at the end of life?”

Editor’s point 3.

“• Have a separate sub-heading for procedures”

This sub-heading appears now before Interview Guide

Editor’s point 4.

“• Reference 27 doesn’t mention ethnographic interviewing (line 170) it mentions grounded theory and phenomenology (but not types) – talking about ethnography may confuse the reader so consider rewording”

The term “ethnographic” has been deleted for clarity.

Editor’s point 5.

“• Have a section on sample size and how you decided when to stop interviewing”

A sentence has been added at the end of the first paragraph of the section entitled “Sample” and a new section entitled “Sample Size and Characteristics” has been added:

The sample allowed the researchers to emphasize the fundamental commonality of informant experience while allowing for variations in order to fully develop themes from the data.

Sample Size and Characteristics

The researchers concluded that substantial saturation of content categories was reached after 33 interviews, at which point interviewing stopped. This was determined by the research team’s high degree of agreement that data that were being consistently coded in specific groups or categories, and that new coding was not repeated significantly. The researchers concluded that substantial saturation of content categories was reached after 33 interviews.

Editor’s point 6.

“• Results seem to appear from around line 196 – 218 – please move to the start of the results”

We could not identify this point by line number.

Editor’s point 7.

“• Please include an audit trail with examples of analysis in a supplementary file readers need to have short example stages that they should go through should they wish to use the approach again - given reference 27's loose consideration to this it is important examples are provided”

A new sentence and a reference to a Supplementary File with a sample of the process of have been added at the end of the “Data Analysis” section:

The process is illustrated with the development of Theme 1 in Supplementary file 2, Progression from sample of interview text to codes, categories, and theme.

A new section entitled “Quality Assurance” with a revised first paragraph have been added after the section entitled “Data Analysis”:

Quality Assurance

Four members of the research team discussed the data and its analysis at the beginning of this stage (Phase 3, Stage 2 from Table 1). Different insights and disagreements in understanding and interpretation were reconciled and the results were agreed upon. In order to strengthen trustworthiness of the findings, the other two team members independently reviewed the data and evolved themes at stage three. While the names of some codes, categories, and themes varied, there was a high degree of agreement on substance. The consistency and compatibility of findings between the data analysts made reconciliation possible with a high degree of concordance. The themes were also presented for discussion by medical professionals during a round-table seminar organized by the research team. Despite a number of digressions based on specific findings, the round-table group concurred that the findings were consistent with the realities that they faced or had experienced, even though they were understood from quite different viewpoints. Table 1. And Supplementary file 2 constitute an audit trail for the data analysis process.

Editor’s point 8.

“• Please include the original and any modified interview script in a supplementary file”

Supplementary File 3 presents a sample from the original transcripts. Full transcripts are available upon request.

Editor’s point 9.

“• Please include a section on how quality was ensured – make sure it has its own sub-heading”

## Covered in conjunction with Editor’s point 7.

We wish to leave Supplementary File 1 (Interview Guide) as it was filed with the original manuscript. We have revised Supplementary File 2, changing the title from “Coded Interview Data” to “Progression from sample of interview text to codes, categories, and theme.” And we have added a Supplementary File 3., entitled “Sample of original interview narrative text.”

We appreciate the opportunity to work with you on the publication and look forward to your response. It is important to note that our funding for publication of the article expires in December 2021, so it would be appreciated if we get your feedback quickly.

Sincerely,

Jolanta Kuznecovienė
